# Interventions to Improve Medication Adherence in Children With Epilepsy: A Systematic Review

**DOI:** 10.7759/cureus.54680

**Published:** 2024-02-22

**Authors:** Karishma Godara, Nisha Phakey, Divyani Garg, Suvasini Sharma, Rashmi R Das

**Affiliations:** 1 Psychology, Jindal School of Psychology & Counselling, O.P. Jindal Global University, Sonipat, IND; 2 Psychology, Chitkara School of Psychology & Counselling, Chitkara University, Rajpura, IND; 3 Neurology, All India Institute of Medical Sciences, New Delhi, New Delhi, IND; 4 Pediatrics (Neurology Division), Lady Hardinge Medical College and Kalawati Saran Children’s Hospital, New Delhi, IND; 5 Pediatrics, All India Institute of Medical Sciences, Bhubaneswar, Bhubaneswar, IND

**Keywords:** convulsion, compliance, pediatric, anti-epileptic drugs, seizure

## Abstract

Low medication adherence remains a major challenge in the treatment of epilepsy, particularly in children. In recent years, several approaches and interventions have been employed to promote medication adherence in children with epilepsy (CWE). In this study, we aimed to summarize the evidence on these interventions. In this systematic review, major medical electronic databases were searched for relevant literature from January 2005 till July 2023, including PsycINFO, Medline (via PubMed), Google Scholar, Taylor & Francis databases, and CENTRAL by the Cochrane Library. We planned to include observational studies (with a control arm) and clinical trials involving children/adolescents (<19 years) with epilepsy and/or their caregivers/families who underwent any intervention to improve adherence to anti-seizure medications. Out of 536 articles searched, eight (six randomized trials and two non-randomized intervention studies) were included in the systematic review. A total of 2,685 children/adolescents along with their caregivers participated in these studies. Six studies used educational and two used behavioral interventions to improve adherence to anti-seizure medications. Four studies showed variable levels of adherence improvement, ranging from 2-20% up to 73.9% post-intervention. To conclude, the findings suggest the potential for educational interventions to promote medication adherence in CWE. The class of evidence was II to III among the included studies, as per American Academy of Neurology guidelines.

## Introduction and background

Epilepsy is a common neurological disorder characterized by recurrent unprovoked seizures affecting individuals across all age groups and socioeconomic cohorts [1,2]. Despite low-cost treatment being available for epilepsy [3,4], low adherence prevails at staggering rates. Adherence rates for anti-seizure medications (ASMs) among children with epilepsy (CWE) have been estimated to range from 22.1% to 96.5% [5]. A meta-analysis concluded a pooled adherence rate of 58% by objective assessment and 73% by subjective assessment measures [5].

Non-adherence to ASMs may potentially result in several adverse outcomes such as poor prognosis, lower health-related quality of life, higher mortality, and higher healthcare costs [6,7]. Medication adherence is defined as “the degree to which the person’s behaviour corresponds with the agreed recommendations from a health care provider” [8]. Medication adherence in pediatric populations is a particularly challenging and complex issue as it involves caregivers, the child, and the clinician. The process depends on a confluence of biological, psychological, and social factors [9]. These factors include the type and severity of the medical condition, socioeconomic status, family’s religious and cultural beliefs, parent’s health literacy, epilepsy knowledge, perceived outcome of treatment, family functioning, and child’s temperament, forgetfulness, and difficulty in swallowing medications [9-12].

Non-adherence is a modifiable variable, and timely behavioral interventions can have a positive impact on medication adherence [2,12]. Given the advances in epilepsy treatment and the availability of low-cost medication, it seems pertinent to identify effective adherence-promoting strategies and interventions. Research on adherence strategies and intervention in people with epilepsy is available. Although a recent Cochrane review of 20 pooled studies encompassing 2,832 participants concluded behavioral interventions as favorable for improving adherence, it focussed mainly on adult cohorts with limited pediatric data [13]. Another review further advocated the need for behavioral and educational interventions to promote medication adherence [6].

This systematic review aims to critically review the extant literature on strategies and intervention models for promoting medication adherence in pediatric epilepsy to assess the efficacy of these interventions and update the literature after the publication of the Cochrane review.

## Review

Methodology

We conducted a systematic review to assess interventions for improving adherence among CWE. This systematic review is reported in accordance with the Preferred Reporting Items for Systematic Reviews and Meta-Analysis (PRISMA) guidelines.

Criteria for Including Studies for Review

We planned to include observational studies (with a control arm) and clinical trials involving children/adolescents (<18 years) with epilepsy and/or their caregivers/families who underwent any intervention to improve adherence to ASMs. Studies included were published between January 1, 2005, and July 31, 2023. No restrictions on sex or ethnicity were employed. Studies that focused on the adult population were excluded.

Search Methodology for Study Identification

The search was conducted using PsycINFO, Medline (via PubMed), Google Scholar, Taylor & Francis databases, and CENTRAL by the Cochrane Library. The following general and Medical Subject Headings (MeSH) keywords were employed for this review: (“medication adherence”) AND ((“children OR child OR youth” AND “epilepsy”) OR “paediatric epilepsy”); (“drug adherence” OR “medication adherence”) AND ((“children OR child OR youth” AND “epilepsy”) OR “paediatric epilepsy)”; (“antiepileptic drug adherence” OR “antiseizure drug adherence” OR “medication adherence” OR “anticonvulsant adherence”) AND ((“children OR child OR youth” AND “epilepsy”) OR “paediatric epilepsy”). Two independent reviewers screened titles and abstracts to identify studies for inclusion. Duplicate articles were excluded. Full-text articles thus identified were accessed and assessed for pre-strategized eligibility criteria, which included children and adolescents and/or their caregivers as the target population for an adherence-promoting intervention and assessed direct or proxy outcome measures of adherence. Studies satisfying these criteria were included in the qualitative analysis.

Data Extraction

Two authors (KG, NP) independently extracted data, including authors, year of publication, study setting (inpatient/outpatient), study design (prospective/randomized trial), participant characteristics (number, age), intervention (type of intervention, duration, schedule), and results (outcome measures). Any disagreement was resolved after discussion with the third author (SS).

Class of Evidence

The class of evidence (CoE) was rated following the American Academy of Neurology guidelines for rating therapeutic studies [14].

Results

**Figure 1:**
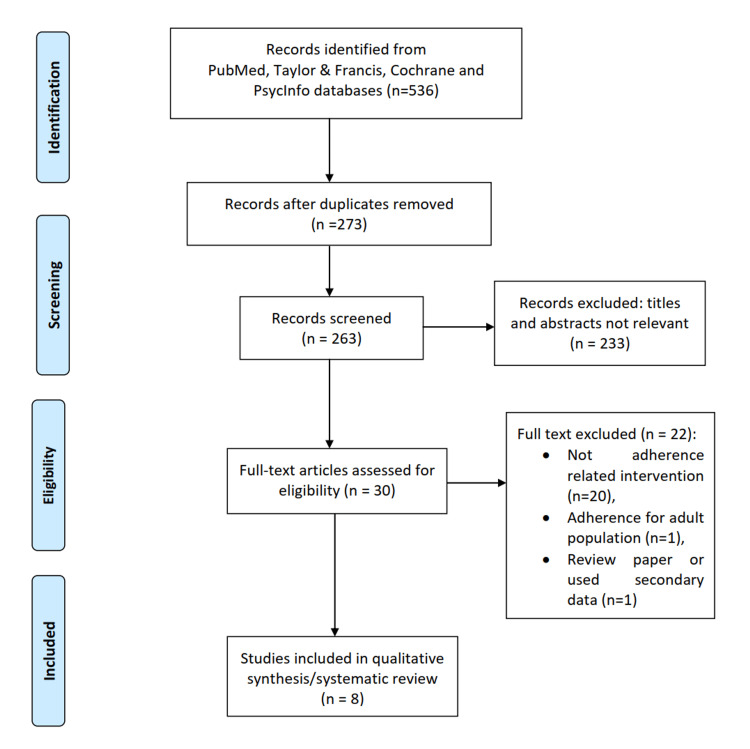
Preferred Reporting Items for Systematic Reviews and Meta-Analysis flow diagram.

The initial search yielded 536 articles, of which 273 articles were excluded because of duplication. After screening titles and abstracts, 30 of 263 articles were assessed for eligibility after accessing the full text. In total, 22 articles were excluded, of which 20 did not involve adherence interventions, one dealt with adults, and one was a review article. Eight articles were finally included in the systematic review (Figure 1) [15-22]. Overall, six were randomized controlled trials, and two were non-randomized intervention studies (Table 1).

**Table 1:** Characteristics of the studies included in the review. AAN: American Academy of Neurology; AI: adherence intervention; ASM: anti-seizure medication; CoE: class of evidence; STAR: supporting treatment adherence regimens; HRQoL: health-related quality of life; MMAS-8: 8-item Morisky Medication Adherence Scale; RCT: randomized controlled trial; TAU: treatment as usual; TDM: therapeutic drug monitoring; VPA: valproic acid; CWE: children with epilepsy

Author (year), country [reference]	Age and number (N) of children	Inclusion criteria	Data collection procedure	Study design and setting	Main results	Limitations
Chen et al. (2013), Singapore [15]	Age: <18 years N: 27	All epilepsy cases commencing treatment, changing ASMs, or non-compliant with treatment	Self-administered questionnaire and pharmacist-collected data through telephonic follow-up after two weeks	Design: Non-randomized intervention study. Setting: A specialist clinic	Educational counseling by the pharmacist was effective at improving caregiver knowledge	Small sample size and short follow-up (two weeks only)
Modi et al. (2013), United States [16]	Age: 2–12 years N: 8	New-onset epilepsy with adherence <90%	Data were collected at baseline, during sessions, and at three to four-month intervals	Design: RCT. Setting: A children’s hospital	The intervention was feasible and acceptable	Small sample and seizure outcomes were not analyzed
Modi et al. (2016), United States [17]	Age: 2–12 years N: 23	Recent diagnosis of epilepsy within seven months	STAR intervention involved one to three assessment visits, four face-to-face and two telephone intervention sessions, and one to three follow-up visits	Design: RCT. Setting: A children’s hospital	Significant improvement was observed in the STAR adherence group	Small sample size, only caregiver-reported questionnaires were used
Modi et al. (2016), United States [18]	Age: 13–17 years N: 25	No other chronic medical disorder that required daily medication	Five intervention groups: text-enabled only versus smartphone	Design: RCT. Setting: A children’s hospital	The text messaging intervention had the highest reported adherence	Baseline adherence was quite high and sample size was small
Saengow et al. (2018), Thailand [19]	Age: 1 month to 15 years N: 214	Those regularly following up at the clinic	Adherence was evaluated before the intervention, immediately after the intervention, and at a three-month follow-up using MMAS-8	Design: RCT. Setting: A pediatric neurology clinic	Significant improvement in adherence scores was observed in the treatment group at three months	Quazi-randomization method and participants were mainly from a rural background
Le Marne et al. (2018), Australia [20]	Age: 13–19 years N: 51	Children referred to the clinic	Clinical outcomes were logged by parents for two weeks at baseline, four weeks during the intervention, and two weeks post-intervention	Design: Prospective study. Setting: hospital network data	The app showed enhanced knowledge acquisition	Small sample and lack of randomization
Ma et al. (2019), China [21]	Age: <14 years N: 2,165	Epilepsy treated with VPA for >1 month and with at least one plasma VPA level measured	Verbal education and written materials were provided by pharmacists. A blood sample for TDM was drawn	Design: RCT Setting: Outpatients from tertiary care teaching hospitals	This intervention improved therapy adherence in CWE	No follow-up evaluation was done
Modi et al. (2021), USA [22]	Age: 2–12 years N: 200	Epilepsy diagnosed in the past seven months, on a single ASM, and baseline adherence <95%	Intervention and control groups underwent eight sessions, six were face-to-face and two were telephonic	Design: RCT Setting: A children’s hospital	This intervention improved therapy adherence in CWE	The study included children with newly diagnosed epilepsy only

Four of the studies were conducted in the United States, and one each in Singapore, Thailand, China, and Australia. Six studies involved children/adolescents and their parents/caregivers as participants. A total of 2,685 children/adolescents along with their caregivers participated in these studies. In the remaining two studies, only families (n = 4) or caregivers (n = 27) of CWE were included. Two studies focussed on adolescents with epilepsy.

Types of Interventions

The main interventional strategy employed in six of the eight studies was educational [15,16,19-22]. The details of the interventions are outlined in Table 2.

**Table 2:** Details of interventions among the studies included in the review. ASM: anti-seizure medication; STAR: Supporting Treatment Adherence Regimens

Author (year) [reference]	Intervention	Intervention administration
Chen et al. (2013) [15]	Educational intervention: Tailored educational pharmacist and counseling pharmacists worked with neurologists to individualize counseling for patients using an already established handbook and hardcopy presentation slides during counseling	Provider: Outpatient pharmacists. Number of sessions: 01. Duration per session: 60 minutes. Duration of intervention: One session only. Medium: Face-to-face
Modi et al. (2013) [16]	Family-tailored education and problem-solving adherence intervention: The intervention encompassed educating the caregiver about epilepsy treatment and ASM adherence, identifying specific barriers to target for change, and working on problem-solving. Further, the intervention included regular feedback and weekly telephonic follow-ups	Provider: Masters-level graduate students and postdoctoral fellows trained by pediatric psychologists. Number of sessions: Four (for two months). Medium: Face-to-face and telephonic
Modi et al. (2016) [17]	STAR: The intervention included addressing deficits in epilepsy knowledge, education about ASM adherence, providing feedback, training for problem-solving, and behavioral contract	Provider: Psychology doctoral students and post-doctoral fellows. Number of sessions: Four (for eight weeks). Medium: Face-to-face and telephonic
Modi et al. (2016) [18]	Text messaging and application-based interventions (5 interventions) 1: Text messaging received by adolescents only. 2: Text messaging received by adolescents and their caregivers. 3: Application for adolescents only. 4: Application for both adolescents and caregivers. 5: The Epilepsy Tool Kit application	Provider: Artificial intelligence (text messaging and/or smartphone applications). Number of sessions: Not applicable. Duration of session: Not applicable. Duration of intervention: 30 days. Medium: Text messages and applications
Saengow et al. (2018) [19]	Educational intervention: A video animation that included six knowledge domains of epilepsy, namely, diagnosis, etiology, treatment, first aid seizure care, prognosis, and safe activity. The content of knowledge in this video was encompassed from the Thai epilepsy guideline	Provider: Clinician. Number of sessions: Single. Duration per session: 8.52 minutes. Duration of intervention: Single point of contact, follow-up at 30 days. Medium: Clinical advice with or without video animation
Le Marne et al. (2018) [20]	Application-based intervention: EpApp was built using adolescent education content used previously in face-to-face education updated with reviews from a multidisciplinary group (pediatric and adult neurologists, psychologists, epilepsy nurses, and epilepsy fellows)	Provider: Artificial intelligence through a smartphone application. Number of sessions: Single. Duration of session: Not mentioned. Duration of intervention: 4 weeks. Medium: A mobile application
Ma et al. (2019) [21]	Educational intervention (intervention hospital): Active patient education and consultation service. The verbal intervention included epilepsy disease state education, detailed information on ASM therapy, and when to contact clinicians	Provider: Pharmacist. Number of sessions: Five. Duration per session: Not mentioned. Duration of intervention: 1 year. Medium: Face-to-face and written education
Modi et al. (2021) [22]	STAR compared to education only intervention STAR: Addressing deficits in epilepsy knowledge, education about ASM adherence, providing feedback, training for problem-solving, and behavioral contract. Education only: Covered a range of topics including seizure safety, sleep hygiene, comorbidities, issues related to school	Provider: Masters and doctoral-level psychologists and trainees. Number of sessions: Eight. Duration per session: 45 minutes (face to face), 15 minutes (telephonic). Duration of sessions: Four months. Medium: Face-to-face and telephonic

Some of the studies did not mention the number of sessions as well as the duration of each session. In one study, a single 60-minute session of counseling was conducted using a handbook and a hard copy of presentation slides [15]. In one study, four sessions were conducted over two months among participants whose adherence was pre-assessed to be below 90% [16]. In another study, four face-to-face and two telephonic problem-solving sessions over eight weeks were compared to usual treatment among families with <95% medication adherence [17]. One study employed text messaging and phone application-based intervention among adolescents with or without caregivers over eight weeks [18]. One study used a video animation of 8.52 minutes on epilepsy in a single-contact session and compared it to a clinician’s advice [19]. One study developed and evaluated a mobile phone-based application (EpApp) as an adherence intervention over four weeks [20]. One study used pharmacist-imparted active education intervention consisting of written and oral material versus standard pharmacists over one year [21].

One study did not employ educational intervention (rather a family-tailored educational and problem-solving intervention) over a four-month period and compared adherence at the 12-month follow-up. A total of eight sessions were conducted in both groups (six in person and two telephonically) [22].

Intervention Providers

Among the eight studies, two involved pharmacists as intervention providers [15,22], three involved psychologists (doctoral fellows and post-doctoral fellows) [16,17,22], one involved the treating clinician [20], and the remaining two involved artificial intelligence (in the form of text messages/smartphone applications) [19,21].

The face-to-face sessions by the outpatient pharmacist in one of the studies were primarily based on educational models and involved only one session for an hour [15]. The tailored interventions encompassing educational and problem-solving components ranged up to four to eight sessions over eight weeks and were administered by trained psychologists [16,17,22]. The application-based interventions had longer assessment periods [19,21].

Outcomes

Adherence was measured by subjective as well as objective measures in the included studies (Table 2). Subjective methods included self-reporting or psychometric tools. Objective methods included electronic measurement methods such as Medication Event Monitoring System (MEMS) TrackCap. Most studies measured psychosocial correlates of adherence such as knowledge, attitude, and perception. Such variables were measured using validated psychometric tools.

Adherence measures: Post-intervention adherence measures were assessed in six of the eight studies. In the preliminary study of family-tailored adherence intervention, two of four families enrolled in the intervention showed a large improvement in adherence rates [16]. However, these families had low baseline adherence rates. One family had a baseline rate of 83% which declined after one month of treatment. The fourth family also showed a decline at one month post-intervention. Three of the four families in the control group showed improvement ranging from 2% to 19%. One family was excluded due to missing post-treatment data.

In the family-tailored problem-solving Supporting Treatment Adherence Regimens (STAR) intervention, children exhibited improved adherence during active intervention compared to the control group, but there were no significant differences between groups during the follow-up period of three months [17]. In another study, text messaging and phone-based application usage among teenagers, with or without caregivers, led to minimal improvement in adherence [18]. Although not statistically significant, this study noted an interesting trend toward decreased adherence levels associated with parental/caregiver involvement.

One study assessing medication adherence employing the 8-item Morisky Medication Adherence Scale (MMAS-8) found a significantly improved adherence at the three-month follow-up in the intervention (video animation) group (42.9%) versus the usual treatment group (15.9%) (p < 0.05) [19]. In another study assessing medication adherence by the Simplified Medication Adherence Questionnaire (SMAQ), adherence was found to improve from a minimum of 56.0% to a maximum of 73.9% and stabilized thereafter during the last six months of follow-up [21].

A non-educational intervention study found significantly better adherence in the intervention (STAR) group (mean: 82.34, standard deviation: 21.29) compared to the control group (education only) (mean: 61.77, standard deviation: 28.29) at the 12-month follow-up, with the STAR group showing 20% increased adherence (p = 0.04) [22].

Electronic monitors such as the MEMS TrackCap or Vaica SimpleMed+pillboxed were used to measure adherence in four studies [16-19,22]. The MEMS measures the date and time when a pill bottle and cap are opened.

Knowledge-based outcomes: Knowledge assessment was the primary outcome in three studies [15,19,20]. In one study, improvement in pre- and two weeks post-intervention scores of caregivers’ knowledge of epilepsy via a questionnaire was established [15]. The response rate was 49%. Mean scores post-counseling were significantly higher than pre-counseling (14.7 versus 10.4; p = 0.000). This study did not directly measure improvement in adherence parameters. Another study assessed the knowledge of epilepsy patients and/or caregivers by employing a 10-item questionnaire created by a pediatric neurologist [19]. Mean scores in the intervention group increased to 7.42 from a baseline of 6.74 immediately after watching the video, and further to 7.47 at the three-month follow-up, versus no change in the control group. In the third study, knowledge acquisition was assessed via a survey [20]. The Adolescent Knowledge of Epilepsy Questionnaire was used to assess general epilepsy knowledge. Both self and general epilepsy knowledge improved post-intervention.

Other outcomes: In one study, there was a significant improvement in caregiver confidence in administering ASMs (3.60 to 3.94; p = 0.002) [19]. The severity of seizures in terms of frequency and duration was also assessed. A higher proportion of patients with improved seizure severity was reported in the intervention (37.3%) versus the control group (25.0%). In another study, psychosocial outcomes were evaluated using the Seizure Self-Efficacy Scale for Children and Adolescents With Epilepsy and the Child Attitude Towards Illness Scale, but the psychosocial outcomes as well as seizure burden did not improve significantly [20]. Serum valproate levels were used as an outcome measure in addition to medication adherence in one study [21]. The percentage of valproate samples reaching the therapeutic range increased post-intervention between the first and the second, third, fourth, and fifth therapeutic drug monitoring measurements. In one study, health-related quality of life and seizure outcomes were assessed, but no difference was noted between the groups at 12 months [22].

Class of Evidence

The CoE was rated as Class II for the included randomized controlled trials (the reason for downgrading by one class was no or unclear allocation concealment/blinding) (Table 3).

**Table 3:** Summary of evidence (for intervention studies) as per the American Academy of Neurology guidelines. RCT: randomized clinical trial; wk: week; mo: month; yr: year; F/U: follow-up; STAR: Supporting Treatment and Adherence Regimens; SMAQ: Simplified Medication Adherence Questionnaire; EO: education intervention; CoE: class of evidence ^a^: Downgraded by one class because of no description of allocation concealment. ^b^: Natural history refers to the adherence to anti-seizure medication that was perfect or improved without receiving any intervention (i.e., they just followed the natural course).

Author (year) [reference]	Sample size	Age	RCT: Yes/ No	Intervention	Medication adherence rate (pre-intervention)	Duration of epilepsy	F/U	Completion rate	Blinding	Class of Evidence (CoE)	Natural history (%)^b^
Chen et al. (2013) [15]	27	Mean: 8.9 yr	No	Questionnaire	Not mentioned	Not mentioned	2 wk	82%	No	III	Not mentioned
Modi et al. (2013) [16]	30	Mean: 7.2 yr	Yes	Questionnaire	Two groups: <90% and ≥90%	7 mo	4 mo	87.5%	Unclear	II^a^	63% (19/30)
Modi et al. (2016) [17]	50	Mean: 7.6 yr	Yes	STAR	Two groups: <95% and ≥95%	7 mo	3 mo	87%	Unclear	II^a^	42% (21/50)
Modi et al. (2016) [18]	25	Mean: 15.7 yr	Yes	Text message, application and communication	Two groups: <95% and ≥95%	7 mo	1 mo	85%	Unclear	II^a^	Not mentioned
Saengow et al. (2018) [19]	214	Mean: 7.6 yr	Yes	Video animation and questionnaire	Intervention group: 52.4% Control group: 54.6%	Not mentioned	3 mo	100%	No	II^a^	0%
Le Marne et al. (2018) [20]	51	Mean: 14.49 yr	No	Mobile application (EpApp)	Not mentioned	Not mentioned	2 wk	76%	No	III	Not mentioned
Ma et al. (2019) [21]	2165	Mean: 5 yr	No	SMAQ	Not mentioned	Intervention group (median): 25.6 mo Control group (median): 22.8 mo	6 mo	100%	Unclear	III	0%
Modi et al. (2021) [22]	200	STAR: 7.13 (2.80) yr EO:8.15 (3.3) yr	Yes	STAR versus EO	STAR: 76.25 (19.3) EO: 72.95 (20.1)	STAR: 2.49 (2.04) EO: 2.63 (2.64)	12 mo	STAR: 22/27 (81.4) EO: 21/29 (72.4)	No	II^a^	Not mentioned

The other three studies were rated as Class III evidence being non-randomized intervention (before and after) studies.

Discussion

Although literature primarily focusing on adherence enhancement among adult populations with epilepsy exists, there is a dearth of such data among the pediatric population [13,23-29]. In this systematic review, eight studies (randomized controlled trials = 6, non-randomized controlled trials = 2) with a total of 2,685 children/adolescents along with their caregivers were included. Six studies used educational and two used behavioral interventions to improve adherence to ASMs. Four studies showed variable levels of adherence improvement, ranging from 2% to 20% that went up to 73.9% post-intervention.

Continued seizures pose a significant challenge among CWE, and a principal factor contributing to this problem is non-adherence to ASMs. Adherence patterns among CWE vary from severe early non-adherence, severe delayed non-adherence, moderate non-adherence, and mild non-adherence to near-perfect adherence [30]. Nearly 58% of children who are newly diagnosed with epilepsy are non-adherent to their ASM prescription over the first six months of treatment [30]. Improving medication adherence is crucial for several reasons: non-adherence correlates with increased seizure frequency, mortality, and expenditure [31,32]. Non-adherence also correlated with seizure outcomes among CWE [33]. Data on measures to improve medication adherence in pediatric epilepsy is limited. In this review, we captured literature on interventions aimed at improving various aspects of medication adherence in CWE.

Most studies included in this review employed various educational interventions alone or as part of multi-component interventions. Educational interventions are considered crucial in health promotion. Such interventions target users’ knowledge, skills, attitudes, confidence, and behavior [34]. Specific barriers contributing to non-adherence toward ASMs among CWE have been explored by Ramsey et al. in a previous study [10]. They identified specific barriers to adherence that remained stable or worsened over a two-year period. Specifically, these included difficulty with swallowing, forgetting to take medications, and the child’s refusal to take medications. It is possible that the latter two barriers may be overcome by appropriate parental or caregiver counseling. However, none of the studies included in the review assessed specific barriers and tailored the intervention to the barriers identified.

Regarding outcome assessment, most studies included in this review used adherence questionnaires. Various direct and indirect measurements of adherence assessment have been proposed. An objective measure is the assessment of ASM level in the blood, as used in one study from China [7]. The disadvantage of this proxy measure of adherence is that blood levels may only indicate that the medication was taken in the previous 48-72 hours and, additionally, measurement of drug levels is often expensive. Additionally, the absence of blood drug levels will inform complete non-adherence alone but a specific drug level will not indicate the level of adherence.

An indirect method is the use of electronic measurement methods such as MEMS, as used in several studies in this review. This method of adherence assessment is usually considered a gold standard for adherence measurement. MEMS is a microchip-enabled system that records a date and time stamp when a pill bottle or package is opened. Adherence is calculated using special software. An intrinsic disadvantage of this method is that opening the pill container may not necessarily be tantamount to the patient having taken the drug. Moreover, this is an expensive technology. Another technique is the use of self-reported adherence questionnaires such as MMAS. Although pragmatic, these measures are subjective. Despite the wide heterogeneity of adherence measures, there is no single one-size-fits-all approach to measure adherence among CWE yet.

Interestingly, although most studies employed educational interventions, the use of behavioral intervention in the form of a short video animation demonstrated improvement in adherence with persisting effects at three months. This may be a useful and simple intervention with a one-time resource investment that may be a practical solution to adherence issues.

The effect of these interventions on non-adherence outcomes such as seizure frequency, quality of life, and optimum drug levels were reported in very limited studies. Hence, the impact of these interventions on CWE remains uncertain. Most studies addressed the impact of adherence interventions for short periods, and their longitudinal impact needs to be assessed. All studies included comprised Class III-IV evidence. There is a notable lack of randomized controlled studies with a larger sample that limits the generalizability of results. Moreover, in adherence intervention assessment, multiple measures of adherence are recommended as there is no single gold standard [13]. None of the studies included in this review have used more than one adherence measure. Hence, there is definite scope for future research with larger sample sizes and longer follow-ups.

Although systematic reviews on drug adherence are available in the current literature [6,13], we could not find any such review focussed on adherence measures among CWE alone. Adherence measures employable among adult persons with epilepsy may not necessarily find extrapolation among CWE.

Limitations

There are a few limitations of this review. The included studies used different interventions to assess improved adherence to ASMs making it difficult to recommend a particular intervention (a meta-analysis could not be done). Long-term follow-up was not done in the included studies (only one study had a six-month follow-up period). The duration of epilepsy was not mentioned in 50% of the studies making it difficult to conclude the utility of educational/other interventions in a particular context. There was no information on difficult-to-control or refractory epilepsy before initiation of intervention making it difficult to provide in these groups of children. As we did not search gray literature, the studies by governments or other agencies using various interventions (with a control arm) might have been missed.

## Conclusions

Given the benefits of medication adherence and the potential risks of non-adherence, state-of-the-art interventions and strategies for this are crucial. The CoE was II to III among the included studies, as per American Academy of Neurology guidelines. The need of the hour is developing tailored interventions and validating them using controlled trials in different population cohorts. Future researchers and clinicians can keep these points in mind while developing interventions and strategies for promoting adherence to ASMs in CWE. An intervention model should integrate strategies and techniques from different schools of therapy, and scientifically validated therapies such as motivational interviewing that are frequently employed for internally motivated behavioral change should be employed. Subjective barriers should be assessed before initiating the intervention, and adherence should be assessed regularly. Children or adolescents should also be encouraged to be part of the intervention process. Importantly, a non-judgmental and collaborative interaction should lead the management process.
